# Association between Mineral Intake and Cognition Evaluated by Montreal Cognitive Assessment (MoCA): A Cross-Sectional Study

**DOI:** 10.3390/nu15214505

**Published:** 2023-10-24

**Authors:** Ana M. Lorenzo-Mora, Ana M. López-Sobaler, Laura M. Bermejo, Liliana G. González-Rodríguez, Esther Cuadrado-Soto, África Peral-Suárez, María Dolores Salas-González, María Luisa Delgado-Losada, Inmaculada C. Rodríguez-Rojo, Ana Barabash, Fernando Maestú-Unturbe, Aránzazu Aparicio

**Affiliations:** 1 Department of Nutrition and Food Science, Faculty of Pharmacy, Complutense University of Madrid, 28040 Madrid, Spain; analor01@ucm.es (A.M.L.-M.); asobaler@ucm.es (A.M.L.-S.); mlbermej@ucm.es (L.M.B.); esther.cuadrado@ucm.es (E.C.-S.); africper@ucm.es (Á.P.-S.); masala06@ucm.es (M.D.S.-G.); araparic@ucm.es (A.A.); 2 VALORNUT Research Group, Complutense University of Madrid, 28040 Madrid, Spain; mldelgad@ucm.es; 3 San Carlos Health Research Institute (IdISSC), 28040 Madrid, Spain; fmaestuu@psi.ucm.es; 4 School of Sport, Exercise and Health Sciences, Loughborough LE11 3TU, UK; 5 Department of Experimental Psychology, Cognitive Processes and Speech Therapy, Faculty of Psychology, Complutense University of Madrid, 28223 Madrid, Spain; 6 Center for Cognitive and Computational Neuroscience, Complutense University of Madrid, 28223 Madrid, Spain; concepcion.rodriguez@uah.es; 7 Department of Nursing and Physiotherapy, Faculty of Medicine and Health Sciences, Universidad de Alcalá, 28871 Madrid, Spain; 8 Faculty of Medicine, Department of Medicine, Complutense University of Madrid, 28040 Madrid, Spain; ana.barabash@salud.madrid.org; 9 Endocrinology and Nutrition Department, Hospital Clínico Universitario San Carlos, Instituto de Investigación Sanitaria del Hospital Clínico San Carlos (IdISSC), 28040 Madrid, Spain; 10 Centro de Investigación Biomédica en Red de Diabetes y Enfermedades Metabólicas Asociadas (CIBERDEM), 28029 Madrid, Spain

**Keywords:** adults, minerals intake, mild cognitive impairment, MoCA

## Abstract

Background: Mineral intake may protect against cognitive impairment (CI) and all-cause dementia, which affects a large number of adults worldwide. The aim of this study was to investigate the association between mineral intake and Montreal Cognitive Assessment (MoCA), which is a sensitive and specific test. Methods: In total, 201 adults were included in a cross-sectional study. They completed a three-day dietary record to estimate their average daily intake of minerals. Contributions to dietary reference intakes (DRIs) were also calculated. The participants were divided into tertiles according to their mineral intake. CI classifications were determined via the MoCA (score < 26). Apolipoprotein E (APOE) genotyping was carried out, and the patients’ anthropometric measurements and physical activity, health and personal data were collected. Results: The prevalence of CI in this selective sample was 54.2% (34.3% females and 19.9% males). In women, being in the third tertiles of iron and manganese intake was associated with lower odds of having CI (OR [95% CI]: 0.32 [0.11 ± 0.93]; 0.33 [0.12 ± 0.93], *p* < 0.05). No significant differences were observed for any of the nutrients studied in men. Conclusions: These findings suggest that a low mineral intake, especially low iron and manganese intake in women, is associated with a worse cognition as assessed by MoCA.

## 1. Introduction

With the increase in the life expectancy of the population in recent decades, there have been significant increases in the incidences of chronic diseases such as cancer and cardiovascular and neurodegenerative diseases [1,2,3]. Specifically, within the latter group, dementia affects about 50 million people worldwide, with about 10 million new cases registered each year [4], making it a public health priority. This syndrome also interferes with occupational, domestic and social functioning [5]. It often begins with mild cognitive impairment (MCI) with which memory loss can appear at an early stage and may trigger the development of Alzheimer’s disease (AD), the most common type of dementia [6].

MCI is characterized by cognitive decline observed subjectively and supported by objective measures compared to a prior level of functioning, which represents the preclinical phase, where healthy aging could lead to dementia. Although there are no approved pharmacological treatments for MCI, progression may be slowed or delayed with focus on reversible causes (such as hypertension, hyperlipidemia, atrial fibrillation and diabetes mellitus) and changing lifestyle (including diet, exercise, tobacco and cognitive stimulation) [7]. The prevalence of MCI in individuals over the age of 65 has been found to range from 10% to 15% [8]. The annual rate of progression to dementia in individuals with MCI is at 5–10%, which is a significantly elevated figure when contrasted with the 1–2% annual incidence rate observed in the general population [7]. Moreover, it has been described that approximately 50% will lead to dementia in 5 years [9]. MCI stage could be an opportunity to apply strategies to delay the progression to dementia [7]. Although some drugs are dispensed to reduce or control some symptoms, there is no pharmacological treatment for slowing or delaying cognitive decline [10].

For this reason, screening for MCI is essential in order to identify modified risk factors in the population, in an attempt to improve cognitive function and delay progression to dementia [4]. However, these possible causative factors are often overlooked and underestimated [7].

Some non-modifiable factors, such as age, sex and genetics, significantly increase the risk of dementia, especially AD. Women are at higher risk than men [11,12,13] and are carriers of the ε4 allele of the apolipoprotein E gene (APOE ε4) [14].

However, there are many factors that are modifiable, and most of them are related to lifestyle such as diet, physical activity, smoking, sleep, obesity, diabetes mellitus, hypertension, hyperhomocysteinemia and others, such as depression, employment status and education level [15]. Therefore, carrying out early, lifestyle-focused interventions in people with MCI could help to prevent the more severe stages of the pathology [16].

Regarding diet, some studies have indicated that it could play an essential role in the prevention and/or delay of dementia [10,17,18]. In this regard, some research has observed associations with some dietary patterns such as the Mediterranean diet, the Dietary Approach to Stop Hypertension (DASH) or the Mediterranean–DASH Diet Intervention for Neurodegenerative Delay (MIND) [19,20,21,22]. Regarding nutrients, several investigations have observed relationships between B vitamins, vitamin C, folates, omega-3 fatty acids and cognitive function [22,23,24,25,26]. Recently, attention has also been paid to the associations between some minerals and cognitive function [27,28,29,30,31], although there is not much information on this topic.

Some studies suggest that certain minerals such as iron, magnesium, copper, zinc, selenium and manganese could be involved in some mechanisms of action related to cognitive function [32,33]. It has been described that these micronutrients play a role in DNA repair, oxidative damage prevention and the correct methylation process of DNA, among other mechanisms [34]. Thus, they appear to be important in the regulation of cell function and neuromodulation and could play a crucial role in antioxidant protection [35]. Furthermore, their antioxidative properties have the potential to mitigate damage induced by free radicals, thereby preventing or retarding the cognitive decline process attributed to the neurotoxic effects produced by the oxidative stress [36].

Therefore, in an attempt to consolidate the scientific evidence, and taking into account that there is hardly any research using the Montreal Cognitive Assessment (MoCA) test [37,38] to measure cognitive function and study its association with diet, the aim of the present study was to assess the relationship between the intake of minerals with described neuroprotective actions and cognitive function in adults, using the MoCA test to categorize participants as either having or not having cognitive impairment.

## 2. Materials and Methods

### 2.1. Participants

This research is part of the project entitled “Cognitive and neurophysiological characteristics of people at high risk for the development of dementia: a multidimensional approach” (COGDEM). This is an observational, cross-sectional study whose objective is to study the physiological characteristics of healthy and pathological aging, with special interest in recruiting individuals at an increased risk of developing AD [39]. The COGDEM cohort consisted of 262 individuals recruited through different channels: day centers for elderly, some professional associations (i.e., telecommunications engineers) and the neurology consultation of the Hospital Clínico San Carlos. Although the COGDEM study is not a case–control study, efforts were made to recruit people at particular risk of developing AD. Therefore, healthy individuals with a family history of AD were encouraged to participate, since they have higher odds of inheriting genes related to cognitive impairment (such as the APOE ε4+ gene) as has been described previously [1]. A team of expert neuropsychologists ensured that the individuals willing to participate met the study’s selection criteria, which have been detailed previously [39,40]. The main inclusion criteria were MMSE ≥ 24, a modified Hachinski score ≤ 4 [41] and a Geriatric Depression Scale Short-Form score ≤ 5 [42]. The main exclusion criteria were: previous history of neurological or psychiatric disorder, medical conditions that have a high risk of associated cognitive symptoms; severe head injury with loss of consciousness within 5 years; any illness indicating a life expectancy of less than 2 years; alcoholism; chronic use of anxiolytics, neuroleptics, narcotics, anticonvulsants or sedative hypnotics; subjects that showed infection, infarction, focal lesions or significant hippocampal atrophy on magnetic resonance imaging (MRI).

**Figure 1:**
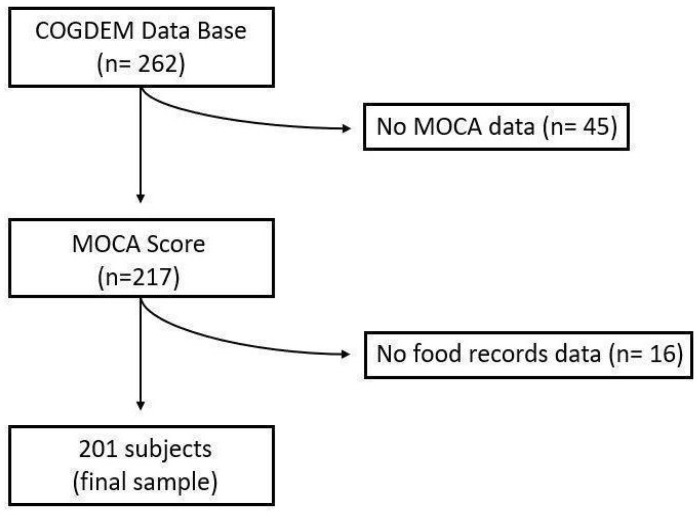
Flow chart of the selection process.

From this cohort, the following subjects were excluded for the present work: individuals who had not completed the MoCA test (*n* = 45) and individuals who had not completed the dietary study (*n* = 16) (Figure 1). Finally, a sample of 201 subjects was included.

All the selected participants signed the informed consent form in order to participate. This research followed the criteria of the Declaration of Helsinki and was approved by the Ethics Committee of the Hospital Clínico San Carlos with the internal code 15/382-E_BS.

The participants underwent a study that examined their health, socio-demographic variables, diet, anthropometric measurements, physical activity, neuropsychological profile and genotype. The study was managed by qualified research staff.

### 2.2. Health and Socio-Demographic Data

Using a questionnaire prepared specifically for this study, the participants were asked about the following factors: (1) their employment status, classifying them as employed, unemployed or retired; (2) their level of education, classifying the population into three groups (whether they received a primary education or lower, a secondary education or a university education); and (3) their use of drugs for hypertension, depression or type 2 diabetes.

### 2.3. Food Record Data

Food and beverage consumption data were collected from a three-day food and beverage consumption record [43] in which participants were required to note all foods and beverages consumed during three non-consecutive days, two during the week and one on the weekend. The dietary data were processed using the nutritional analysis software DIAL [44], which uses data from the Spanish food composition tables [45]. For the present study, data for the following nutrients were analyzed: energy (kcal/day), iron (mg/day), magnesium (mg/day), copper (µg/day), selenium (µg/day), zinc (mg/day) and manganese (mg/day). The studied minerals were adjusted for energy intake via the Willett residual model [46].

Then, the mineral contributions were calculated using the dietary reference intakes (DRIs) established by the Institute of Medicine (IoM), which provided the estimated average requirement (EAR) for all the nutrients studied except for manganese, for which the adequate intake (AI) was used [47,48,49].

### 2.4. Anthropometric Data

Data on the weight, height and waist, hip and calf circumferences of the participants were used for the present study. The anthropometric data were collected according to ISAK guidelines [50], with the subjects standing barefoot and unclothed in a relaxed position.

Each participant’s weight (kg) was measured using a Tanita Body Fat Monitor Scale, White Backlit LCD Display model UM-017 (range: 0.1–150 kg; precision: 100 g), which is an electronic, digital scale, and the height (cm) of each participant was obtained using a Harpenden digital stadiometer (range 70–205 cm; precision: 1 mm). With these two measurements, the subjects’ BMI values were calculated using the following formula: weight (kg)/height^2^ (m^2^) [51].

The waist and hip circumferences were determined to evaluate possible cardiovascular risk [52], and the calf circumference was determined to establish the presence of sarcopenia [53]. All circumferences were measured using a HOLTAIN steel tape measure (range: 0–150 cm; accuracy: 1 mm).

### 2.5. Physical Activity

Physical activity data were recorded via ActiGraph wGT3X-BT accelerometers (Pensacola, FL, USA). The participants wore the accelerometer on the right hip for 7 days, and finally, data from those who recorded more than 10 h per day on at least 4 days of the week were taken, with a minimum of one of those days falling on the weekend [54,55,56,57]. ActiLife software (6.13.3) (LLC, Pensacola, FL, USA) was used to collect physical activity data of the participants. To classify the intensity of physical activity, the following criteria were applied: sedentary time (<100 counts/min); light activity (100–1951 counts/min); and moderate to vigorous physical activity (MVPA) (≥1952 counts/min) [58].

### 2.6. *APOE* Genotyping

Blood samples of 10 mL were extracted in ethylenediaminetetraacetic acid (EDTA) tubes to obtain the genomic DNA. To carry out APOE genotyping (i.e., rs7412 and rs429358 polymorphisms), TaqMan assays were conducted on an Applied Biosystems 7500 Fast Real Time PCR machine. As a result, the participants were classified as carriers (APOE ε4+) or non-carriers (APOE ε4−) of the ε4 allele of the APOE gene.

### 2.7. Neuropsychological Test

#### 2.7.1. Geriatric Depression Scale (GDS)

The 15-item GDS [42] was used for the study of depression. Each question is answered dichotomously (yes/no), which is scored as 1 or 0, respectively, with a maximum score of 15 points. Scores above 5 probably indicate depression symptomatology.

#### 2.7.2. Mini-Mental State Examination (MMSE)

Mental status was also evaluated by means of the MMSE test, which is composed of different areas: spatial and temporal orientation, immediate memory, attention and calculation and delayed memory and language [59].

#### 2.7.3. Montreal Cognitive Assessment (MoCA)

The MoCA is a cognitive screening tool to assist in detection of mild cognitive impairment (MCI) [37]. This test has been validated for the Spanish population [60]. This test studies different abilities such as attention, concentration, memory, language and executive functioning.

The test is structured as follows: (1) visuo-spatial abilities are assessed by having the subject draw a clock (3 points) and copy a three-dimensional cube (1 point); (2) executive function is assessed by having the subject complete an alternation task adapted from the Trail Making B task (1 point), a phonemic fluency task (1 point) and a two-item verbal abstraction task (2 points); (3) attention, concentration and working memory are assessed by having the subject complete a sustained attention task (target detection by tapping; 1 point), a serial subtraction task (3 points) and assess forward and backward digits (1 point each); (4) short-term recall is assessed by having the subject complete two trials involving learning nouns and their delayed recall after five minutes (5 points); (5) language is assessed by having the subject complete a three-item naming task with animals (3 points), repeat two syntactically complex sentences (2 points) and complete the fluency task mentioned above; and (6) the subject’s orientation in time and place is assessed (6 points).

The maximum achievable score is 30, and scores below 26 suggest MCI [37]. In our study, since we do not have a clinical diagnosis of MCI we have used this cut-off point to assess CI.

### 2.8. Statistical Analysis

Data for continuous variables are expressed as means and standard deviations. Percentages were also calculated for the qualitative variables studied. Dietary and anthropometric data were compared according to sex and according to the MoCA score (CI group: MoCA score < 26; non-CI group: MoCA score ≥ 26).

For the comparison of means, the Mann–Whitney U test and Student’s *t*-test were used for variables following a non-normal or normal distribution, respectively. A two-way ANOVA test was used to determine the relationships between energy and mineral intake (quantitative dependent variable) and the MoCA score and sex (qualitative independent variables). A Z-test was used to determine the differences between proportions. Spearman’s correlation was applied since the scores obtained via the MoCA test did not follow a normal distribution.

Tertiles of consumption of each mineral were calculated. The association between the tertile of the intake of each mineral (independent variable) and MoCA score (dependent variable) was analyzed via logistic regression to calculate odds ratios (ORs). Mineral intake was calculated with a 95% confidence interval. Crude ORs were calculated (model 1), additionally corrected for age and BMI (model 2) and further corrected for educational level, employment status, drug intake, physical activity, family history of Alzheimer’s disease, APOE genotype and depression (model 3). The statistical significance level was set at *p* < 0.05.

## 3. Results

A total of 201 subjects were included (63.2% female), with a mean age of 59.8 ± 7.9 years (from 41 to 81 years old). Of the total sample, 54.2% (34.3% females and 19.9% males) presented scores lower than 26 points in the MoCA test.

Table 1 shows the general characteristics of the sample of the present study: personal and health data, anthropometric data, physical activity, APOE genotyping and the scores obtained from the neuropsychological tests.

In general, with respect to health data (Table 1), for the total sample, it can be observed that the percentage of subjects who studied at a university was higher in the non-CI group than in the CI group. In the latter group, a higher percentage of people with either a primary level of education or no education was found. This difference was also observed when sex was taken into account.

For anthropometry, physical activity, family history of Alzheimer’s disease, APOE genotype and the scores obtained from the depression assessment tests via the GDS, no significant differences were observed according to the MoCA score.

No significant differences were observed in the MMSE according to MoCA score, nor for the total sample according to sex.

The data regarding energy and mineral intake according to the CI and sex are shown in Table 2.

Significant differences were found in the contributions to the DRIs of iron and manganese for the total sample and for women, having been found to be lower in the CI group than the non-CI group (Table 2).

Positive correlations were also observed for the total sample between the MoCA score and copper intake (Rho: 0.189; *p* = 0.007), iron contribution (Rho: 0.174, *p* = 0.014), manganese contribution (Rho: 0.220 *p* = 0.002) and copper contribution (Rho: 0.212, *p* = 0.003).

In women, positive correlations were also observed between the MoCA score and copper intake (Rho: 0.259, *p* = 0.003), manganese intake (Rho: 0.178, *p* = 0. 045), iron contribution (Rho: 0.218 *p* = 0.013), magnesium contribution (Rho: 0.177 *p* = 0.047), copper contribution (Rho: 0.331 *p* = 0.000) and manganese contribution (Rho: 0.310 *p* = 0.000).

No significant differences were observed for any of the nutrients studied in men.

When analyzing the MoCA score according to the tertiles of mineral intake (Table 3), it was observed that those in the total sample with intakes of magnesium, copper and manganese in the first tertile obtained lower MoCA scores compared to those in higher tertiles.

In women, those with intakes of iron, magnesium, copper and manganese in the first tertile had lower MoCA scores than those in higher tertiles. However, no significant differences were observed in men.

In general, the results show that all persons in T1 for the total sample for any of the nutrients evaluated, as well as for women and men separately, had mean MoCA scores below 26 points.

Table 4 and Table 5 show the association between mineral intake and the presence of CI, as measured by the MoCA, in women and men. The analysis was corrected for different covariates that influence the development of CI (age, BMI, employment status, educational level, drug intake, physical activity, family history of Alzheimer’s disease, genetics and depression).

Women with an iron intake within the third tertile (>15.37 mg/day) were less likely to have CI than those with an iron intake within the first tertile (<13.47 mg/day). The same was true for manganese: women in the third tertile (>3.10 mg/day) were less likely to have CI than those in the first tertile (<1.82 mg/day).

## 4. Discussion

The present study investigated the association between the intake of minerals with neuroprotective actions and cognitive function, which was measured via a highly sensitive test for the screening of CI, the MoCA test, in a cohort of Spanish adults. People with CI have lower contributions to the DRIs of iron and manganese, especially women. People in the lowest tertile for magnesium, copper and manganese intake achieved lower scores on the cognitive test, and being in the highest tertile for iron and manganese intake was associated with higher MoCA score in women.

Although AD has been hypothesized to be impacted by copper deficiency [61], studies that have examined the association between copper intake and cognitive function show conflicting results. Some studies observed that compared to those with lower intakes, adults with higher copper intakes have a decreased risk of low cognitive test scores [15] or a slower progression of cognitive decline [62]. However, other studies suggest that a higher copper intake may be associated with worse cognitive ability [63], especially when combined with a high intake of saturated fat and trans fats [64]. On the other hand, research showing an association between elevated serum copper levels and lower cognitive function has been questioned as they may actually be indicators of copper intoxication and do not adequately reflect the mineral status [61].

In the NHANES study, a positive relationship between the intake of copper and iron intake and cognitive ability was observed [15]. However, in a systematic review conducted by Loef and Walach, when analyzing the results of the included clinical trials, no relationship was found between cognitive ability and copper or iron intake [65].

Our study found that in women, iron intakes in the third tertile (>15.37 mg/day) were associated with higher MoCA scores and a lower likelihood of CI. These results are similar to those found in the PATH Through Life Project study [66], which found that for females, those who consumed more iron had a lower risk of MCI. However, contrary to our study, the PATH Through Life Project observed that in men, a high iron intake was associated with a higher risk of MCI. The authors suggested that these differences may possibly be due to physiological differences or to the overall diet, physical activity or general health [66]. The physiological differences may explain the results found in our study since when analyzing diet, physical fitness and general health according to sex, no differences were observed between the groups. In addition, Vercambre et al. [67] did not find association between iron intake and cognitive decline or functional impairment by instrumental activities of daily living (IADLs), but the mean intake of iron in this population was below the mean for women in this study.

Iron is an important nutrient for brain metabolism. Lower levels of this mineral can lead to impaired neurotransmitter regulation, reduced myelin production, changes in synaptogenesis and decreased function of basal ganglia [68]. Moreover, this deficiency could cause anemia, which is quite common in the elderly and is related to decreases in physical, functional and cognitive capacity [69]. Nevertheless, high iron blood levels could be related to pro-oxidant effects [70], so it is important to correctly monitor both parameters. It would be necessary to carry out more studies to further elucidate the relationship of iron and cognitive function.

In a systematic review that analyzed the roles of copper and iron in cognitive ability, it was shown that excessive intakes of iron and copper, combined with a diet high in saturated fatty acids, may have adverse effects in people at risk of AD [65].

In this regard, Morris et al. [64] found a higher incidence of cognitive decline in individuals with increased intakes of copper and with higher intakes of saturated and trans fats but not in those with higher intakes of copper and lower intakes of saturated and trans fats. It has been stated that this could be because these types of fats increase blood cholesterol levels, which could favor the formation and progression of beta-amyloid plaques in the brain; however, an excess of copper could promote the oxidation of fat-originating compounds that could be neurotoxic, therefore contributing to the accumulation of beta-amyloid plaques [71]. However, in our study, when analyzing the probability of presenting CI according to saturated and trans fat intakes, no associations were found.

Manganese is an essential micronutrient that is necessary for different functions as a coenzyme in numerous biological processes involved in the maintenance of cognitive function, which include energy metabolism, antioxidant systems, brain ammonia clearance and the synthesis of neurotransmitters [72]. However, in patients using total parenteral nutrition with high levels of this mineral, cognitive problems have been described, causing manganese levels to be reduced in nutritional formulas. Nevertheless, when the intake of this mineral from food is high, plasma manganese levels are autoregulated by increased metabolism for pancreatic and biliary excretion [73,74,75].

In our study, higher manganese intake was associated with lower odds of having CI. In a cross-sectional study conducted with 6863 participants and after adjusting for several variables, no association was found between manganese intake and cognitive capacity, which was explained by the fact that most participants’ intakes were below the DRIs [63], so an adequate dietary intake of the mineral may contributes to successful aging; it may not only help sustain a healthy body composition and fitness but possibly also prevent age-related disorders such as depression, poor cognition, cardiovascular disease and diabetes mellitus [76].

This mineral plays an important role in brain development and adequate cognitive function [77]. Although manganese can be found in the body in reduced (Mn^2+^) and oxidized (Mn^3+^) states, usually only a small amount of manganese is found in oxidized form. In the literature, it has been described that when Mn^3+^ accumulates, it can trigger neurotoxic processes and may affect cognitive function [78,79]. In our study, although blood data for this mineral were not available, it was found that women had higher levels of intake of other antioxidant nutrients than men, which could explain the differences found according to sex.

Regarding magnesium, this mineral is essential for neuronal transmission and plays a key role in the major excitatory and inhibitory neurotransmission pathways [80]. Thus, Ozawa et al. demonstrated that dietary intake of magnesium was associated with lower risks of all-cause dementia [81] and other studies have found a positive association between cognitive function and the intake of this mineral, and it has been proposed that this mineral has neuroprotective effects, such as the ability to increase cerebral blood flow [82]. In our study, in women, magnesium intakes between 296.19 and 356.62 mg/day were associated with better cognition (i.e., less likely to have CI). However, Vercambre et al. [67] did not find an association between magnesium intake and recent cognitive decline or IADL in the E3N cohort which included 98,995 French women.

Regarding the other minerals explored in this study, such as zinc and selenium, the results published to date are mixed. Selenium is involved in central nervous system function, such as memory or cognitive capacity, and its deficiency has been associated with an increased risk of cognitive decline, impairment of the immune system and mortality [83,84,85].

Thus, in our study, no significant differences were found between the levels of intake of these minerals and cognitive impairment, which coincides with the results of Lo et al. [29], Wang et al. [86] and Bojar et al. [82]. As for zinc, inverse associations have been found between its intake and cognitive function [87,88], and it has been indicated that zinc is a nutrient involved in decreases in beta-amyloid adhesiveness and in the synthesis of amyloid precursor protein [89].

### Limitations and Strengths

Several limitations of our study are noteworthy. First, as a cross-sectional study, it was not possible to infer the causal association of mineral intake with cognition. Second, the participants were recruited from hospital neurology practices and professional associations, presenting a risk of participation bias. In addition, having selected part of the sample through a hospital may imply a higher frequency of risk factors such as diabetes, hypertension and depression in the participants, although the data were corrected for these variables. Third, the pathologies (with the exception of depression) present in the participants were self-reported, which could be a reporting bias. Fourth, the lack of fasting blood data did not allow for a biomarker study. Fifth, participants were recruited only in the Community of Madrid and it was a selective sample since the participants were at high risk of developing AD, so the results may not be generalizable to other populations. Sixth, this study used the MoCA cut-off score (<26) to assess CI, which is the same cut-off used for MCI. Nevertheless, as in the present study a clinical diagnosis of MCI has not been carried out, it is not possible to establish a true diagnosis of MCI. Therefore, the data should be interpreted with caution.

Despite these limitations, the current study also has its strengths. First, the MoCA was used to assess CI. According to several authors, this test has higher levels of sensitivity and specificity than the MMSE in detecting MCI (83–75% vs. 71–74%, respectively) [38,90]. In fact, in our study, which included people with an MMSE score ≥ 24 (meaning that they did not present cognitive impairment), when classified according to the MoCA, it was found that 54.2% (34.3% of women and 19.9% of men) of the participants presented CI. Other studies also showed that MoCA could be superior to MMSE in discriminating between individuals with MCI and without MCI [90]. MMSE and the MoCA test are the most commonly used screening methods in clinical and research fields. Despite this, the MoCA test has shown differences in cognitive profile even in those individuals who were in the normal range on the MMSE. So, the MoCA test would appear to be a useful brief tool to screen MCI, particularly where the ceiling effect of the MMSE may be a problem [91].

The second advantage is the use of a 3-day food and beverage consumption record in order to obtain the average nutrient intake in the population. The main advantage was the collection of accurate quantitative information on individual intake during the registration period, which provided a high level of specificity for different meals. Moreover, the questionnaire did not rely on the respondent’s memory since information was recorded at the time of consumption. This aspect is very important in populations with cognitive decline [92,93].

The last strength of this study is that it took into account some covariables that could influence CI, such as age, BMI, employment status, educational level, drug consumption, physical activity, family history of Alzheimer’s disease, APOE genotype and the presence of depressive symptomatology. Among these factors, we would like to highlight that 59.2% of our sample had undertaken university studies; in fact, this aspect influenced the choice of a cut-off MoCA score < 26 for assessing a possible MCI since some authors [94,95,96] indicate that this aspect should be taken into account when establishing the cut-off point. Other authors use lower cut-off points, which would mean an underestimation in the determination of the prevalence of MCI. Another cofactor that we took into account that other authors do not include is the APOE genotype [97,98], although in our studies we have not observed significant differences depending on whether an individual is or is not a carrier of the ε4 risk allele. Our study allows us to evaluate its influence on the obtained results since it was considered in the logistic regression models. Finally, among the covariates, we would like to highlight that, to our knowledge, no study has included the presence of depressive symptomatology, an aspect which is a key factor in the development of MCI [99,100,101].

## 5. Conclusions

The intake of minerals with neuroprotective actions, such as iron and manganese, could play an important protective role against CI, especially in women, since the higher the intake of these minerals the lower the odds of having CI. The lack of association in the male population could be due to physiological differences and to the fact that they generally contributed less to the DRIs of the minerals studied.

Therefore, this study highlights the importance of the study of mineral intake in the general population and in groups at greater risk in order to avoid low intake levels that could be associated with a worse cognitive capacity assessed by MoCA.

Intervention and follow-up studies monitoring dietary intake and nutritional status (including biochemical parameters) are needed to confirm the possible protective effect of iron and manganese intake on cognitive impairment and to take a deeper look at the differences found in these associations between mineral intake and cognitive function according to sex.

## Figures and Tables

**Table 1:** General characteristics of the sample according to the MoCA score and sex.

	Total		Women		Men	
	Non-CI	CI	Non-CI	CI	Non-CI	CI
*n*	92	109	58	69	34	40
Age (X ± SD)	58.6 ± 7.9	60.7 ± 7.8	58.5 ± 7.3	60.1 ± 7.7	58.9 ± 8.8	61.7 ± 7.9
Family history of Alzheimer’s disease (%)	70.7	66.9	72.4	69.5	67.6	62.0
**Employment status (%)**						
Employed	64.1	50.9	62.1	54.4	67.6	45.0
Unemployed	9.7	13.9	13.8	20.6	2.9	2.50
Retired	26.0	35.1	24.1	25.0	29.4	52.5
**Level of education (%)**						
Primary education or lower	17.4	31.2 *	15.5	33.3 *	20.6	27.5
Secondary education	10.9	20.9	12.1	14.5	8.8	30.0 *
University education	71.7	48.6 *	72.4	52.2 *	70.6	42.5 *
**Drug intake (%)**						
Antihypertensives	16.3	14.7	13.8	14.5	20.6	15.0
Antidepressants	3.3	8.3	3.4	7.2	2.9	10.0
Antidiabetics	3.2	3.7	5.1	2.9	0.0	5.0
**Anthropometric data (X ± SD)**						
Weight (kg) **S**	70.5 ± 13.5	70.7 ± 14.7	64.1 ± 10.2	64.3 ± 11.1	81.4 ± 11.5	81.8 ± 13.7
Height (cm) **S**	164.9 ± 8.8	163.1 ± 9.0	160.5 ± 6.7	158.0 ± 5.9	172.6 ± 6.2	171.9 ± 6.1
BMI (kg/m^2^) **S**	25.8 ± 4.2	26.5 ± 4.4	24.9 ± 4.5	25.8 ± 4.3	27.3 ± 3.4	27.6 ± 4.2
Waist circumference (cm) **S**	86.2 ± 12.7	87.8 ± 13.1	80.2 ± 10.6	82.2 ± 10.8	96.4 ± 8.8	97.5 ± 10.8
Hip circumference (cm)	101.12 ±6.8	100.8 ± 8.3	100.54 ± 7.6	100.4 ± 9.0	102.1 ± 4.9	101.6 ± 7.0
Calf circumference (cm) **S**	36.7 ± 2.7	36.5 ± 3.3	35.8 ± 2.6	35.4 ± 2.8	38.3 ± 2.2	38.3 ± 3.3
**Physical activity (%)**						
**Moderate intensity**						
<150 min/week	36.8	30.0	35.2	30.2	39.4	29.7
150–300 min/week	40.2	37.0	44.4	42.9	33.3	27.0
>300 min/week	22.9	33.0	20.4	26.9	27.3	43.2
**Vigorous intensity**						
<75 min/week	97.7	99.0	98.1	98.4	96.9	100.0
75–150 min/week	2.3	0.0	1.8	0.0	3.0	0.0
>300 min/week	0.0	1.0	0.0	1.6	0.0	0.0
***APOE*** **genotype (%)**						
APOE ε4−	64.1	76.1	62.1	71.0	67.6	85.0
APOE ε4+	35.9	23.8	37.9	28.9	32;3	15.0
**Neuropsychological tests (X ± SD)**						
GDS (score)	1.2 ± 1.8	1.3 ± 1.7	1.2 ± 1.5	1.2 ± 1.5	1.3 ± 2.1	1.4 ± 1.9
MMSE (score)	29.0 ± 1.3	28.8 ± 1.2	28.9 ± 1.5	28.8 ± 1.2	29.1 ± 1.1	28.9 ± 1.3
MoCA (score) **M**	28.5 ± 1.1	23.9 ± 2.1 *	28.6 ± 1.2	23.9 ± 2.2 *	28.3 ± 1.1	23.8 ± 1.9 *

Non-CI—no cognitive impairment; CI—cognitive impairment; X—mean; SD—standard deviation; BMI—body mass index; GDS—Geriatric Depression Scale; MMSE—Mini-Mental State Examination; APOE—Apolipoprotein E; MoCA—Montreal Cognitive Assessment. Two-way ANOVA analysis: **S**—differences according to sex; **M**—differences according to MoCA. For a comparison of means, the Mann–Whitney U test and Student’s *t*-test were used for variables following a non-normal or normal distribution, respectively. Associations between categorical variables were analyzed with the χ^2^ test and a Z-test of proportions. * *p* < 0.05 with respect to non-CI.

**Table 2:** Energy and mineral intake according to MoCA score and sex.

	Total		Women		Men	
	**Non-CI**	**CI**	**Non-CI**	**CI**	**Non-CI**	**CI**
*n*	92	109	58	69	34	40
**Intake**						
Energy (kcal/day) **S**	2089 ± 449	2067 ± 539	1999 ± 423	1928 ± 465	2243 ± 457	2308 ± 577
Iron (mg/day) **S**	15.5 ± 3.3	15.39 ± 6.22	15.1 ± 3.0	14.5 ± 2.8	16.3 ± 3.6	16.9 ± 9.4
Magnesium (mg/day)	332.3 ± 65.5	334.8 ± 88.7	340.0 ± 66.1	333.0 ± 73.6	319.22 ± 63.3	337.93 ± 111.1
Copper (µg/day)	2476.1 ± 765.8	2393.0 ± 681.3	2493.9 ± 608.8	2341.2 ± 521.2	2445.6± 987.7	2481.5 ± 893.9
Zinc (mg/day)	10.6 ± 3.1	10.6 ± 3.4	10.2 ± 2.1	10.6 ± 3.4	11.3 ± 4.3	10.7 ± 3.5
Selenium (µg/day)	112.8 ± 39.0	109.3 ± 32.7	110.0 ± 32.8	108.1 ± 26.9	117.7 ± 48.0	111.4 ± 41.1
Manganese (mg/day)	2.7 ± 1.8	2.7 ± 2.0	2.9 ± 1.7	2.6 ± 1.8	2.5 ± 1.9	2.89 ± 2.4
**Contribution**						
Energy (% EAR) **S**	99.9 ± 21.3	99.4 ± 23.8	103.3 ± 22.1	100.9 ± 23.3	94.0 ± 18.5	96.7 ± 24.8
Iron (% EAR)	287.01 ± 72.9	277.1 ± 120.6 *	285.9 ± 72.8	261.5 ± 74.7 *	289.0 ± 74.0	303.9 ± 171.4
Magnesium (% EAR) **S**	114.4 ± 31.4	113.3 ± 35.4	124.6 ± 29.9	119.1 ± 34.4	96.9 ± 25.8	103.3 ± 35.2
Copper (% EAR)	255.1 ± 85.1	265.9 ± 75.7	277.1 ± 67.6	260.2 ± 57.9	271.7 ± 109.7	275.7 ± 99.3
Zinc (% EAR) **S**	132.01 ± 41.8	131.5 ± 53.3	138.0 ± 37.7	140.1 ± 56.5	121.8 ± 46.7	116.7 ± 44.1
Selenium (% EAR) **S**	252.5 ± 95.8	242.6 ± 87.3	237.56 ± 77.73	226.5 ± 73.6	277.9 ± 117.4	270.3 ± 101.9
Manganese (% AI)	199.0 ± 185.2	153.6 ± 69.8 *	216.4 ± 196.1	152.7 ± 63.2 *	169.4 ± 163.6	155.3 ± 81.1

MoCA—Montreal Cognitive Assessment; Non-CI—no cognitive impairment; CI—cognitive impairment; EAR—estimated average requirement; AI—adequate intake. Two-way ANOVA analysis: **S**—differences according to sex. Significant differences were determined via the Mann–Whitney U test or Student’s *t*-test, as appropriate. Nutrients were adjusted for energy intake via Willett’s method of residuals. * *p* < 0.05.

**Table 3:** MoCA score according to mineral intake tertiles and sex.

	**Total**			**Women**			**Men**		
	**T1**	**T2**	**T3**	**T1**	**T2**	**T3**	**T1**	**T2**	**T3**
**Iron** (median mg/day)	12.5	14.7	18.0	12.4	14.5	17.5	12.4	15.7	20.0
MoCA (score) **I**	25.6 ± 3.1	25.7 ± 2.7	26.6 ± 2.5	25.8 ± 3.1	25.1 ± 2.8	27.1 ± 2.7 **b**	25.2 ± 3.2	26.5 ± 2.4	25.8 ± 2.4
**Magnesium** (median mg/day)	268.7	321.7	395.8	276.6	325.3	400.0	256.58	314.9	318.8
MoCA (score)	25.3 ± 3.3	26.5 ± 2.5 **a**	26.2 ± 2.5	25.1 ± 3.3	26.9 ± 2.5 **a**	26.2 ± 2.6	25.7 ± 3.3	25.8 ± 2.4	26.1 ± 2.4
**Copper** (median µg/day)	1950.1	2270.9	2790.8	1980.7	2295.9	2795.0	1880.2	2250.4	2780.5
MoCA (score) **T**	24.9 ± 3.2	26.5 ± 2.6 **a**	26.6 ± 2.4 **a**	24.8 ± 3.3	26.4 ± 2.6 **a**	26.9 ± 2.4 **a**	25.1 ± 3.1	26.5 ± 2.7	25.6 ± 2.1
**Zinc** (median mg/day) ‡	8.5	10.2	12.2	8.6	10.1	11.74	8.4	10.3	12.8
MoCA (score)	25.6 ± 3.1	26.0 ± 2.8	26.3 ± 2.7	25.9 ± 3.2	26.0 ± 2.6	26.3 ± 2.9	25.2 ± 2.8	26.0 ± 3.1	26.4 ± 2.2
**Selenium** (median µg/day)	80.7	108.9	135.1	82.0	109.8	129.3	75.1	107.3	152.2
MoCA (score)	25.9 ± 3.1	26.2 ± 2.7	25.9 ± 2.8	25.93 ± 3.32	26.55 ± 2.62	25.74 ± 2.79	25.80 ± 2.68	25.64 ± 2.78	26.21 ± 2.86
**Manganese** (median mg/day) ‡	0.9	2.5	4.3	1.0	2.5	4.2	0.6	2.4	4.6
MoCA (score) **T**	25.1 ± 3.3	26.7 ± 2.3 **a**	26.2 ± 2.7	25.0 ± 3.3	26.5 ± 2.6 **a**	26.7 ± 2.6 **a**	25.3 ± 3.3	26.9 ± 1.9	25.5 ± 2.6

MoCA—Montreal Cognitive Assessment; Data are shown as means ± standard deviations. ‡ Minerals not covered by DRIs (EAR of magnesium: 350 mg/day in men; EAR of zinc: 9.4 mg/day in men; IA of manganese 2.3 mg/day in men and 1.8 mg/day in women). For a comparison of means, the Kruskal–Wallis test was used because the distribution of all variables was not normal, and a two-way ANOVA analysis was used for the following: **I**—interaction between sex and mineral intake; **T**—differences by tertile of mineral intake. Significant pairwise differences are indicated by letters and bold type (**a**—differences from T1; **b**—differences from T2, *p* < 0.05).

**Table 4:** Association between mineral intake and MoCA in a female population. Logistic regression analysis.

	Model 1		Model 2		Model 3	
	OR (95% CI)	*p*	OR (95% CI)	*p*	OR (95% CI)	*p*
**Iron**						
Tertile 1	1		1		1	
Tertile 2	1.606 (0.66–3.92)	0.298	1.514 (0.61–3.74)	0.368	2.437 (0.79–7.50)	0.120
Tertile 3	0.400 (0.17–0.96)	**0.040**	0.334 (0.13–0.85)	**0.021**	0.326 (0.11–0.94)	**0.037**
**Magnesium**						
Tertile 1	1		1		1	
Tertile 2	0.594 (0.25–1.41)	0.236	0.606 (0.25–1.45)	0.260	0.570 (0.21–1.56)	0.549
Tertile 3	0.791 (0.33–1.87)	0.595	0.739 (0.30–1.82)	0.511	0.767 (0.26–2.22)	0.625
**Copper**						
Tertile 1	1		1		1	
Tertile 2	0.711 (0.30–1.68)	0.436	0.711 (0.30–1.69)	0.439	0.580 (0.21–1.60)	0.293
Tertile 3	0.485 (0.20–1.17)	0.106	0.499 (0.21–1.21)	0.124	0.569 (0.20–1.59)	0.282
**Zinc**						
Tertile 1	1		1		1	
Tertile 2	0.720 (0.31–1.69)	0.452	0.688 (0.29–1.64)	0.399	0.619 (0.22–1.79)	0.375
Tertile 3	0.872 (0.37–2.06)	0.754	0.861 (0.36–2.05)	0.736	0.754 (0.28–2.05)	0.580
**Selenium**						
Tertile 1	1		1		1	
Tertile 2	0.655 (0.28–1.54)	0.332	0.596 (0.24–1.45)	0.255	0.531 (0.19–1.53)	0.240
Tertile 3	0.960 (0.41–2.27)	0.926	0.917 (0.38–2.19)	0.845	0.832 (0.30–2.29)	0.722
**Manganese**						
Tertile 1	1	1	1			
Tertile 2	0.439 (0.18–1.06)	0.066	0.450 (0.19–1.09)	0.077	0.477 (0.17–1.32)	0.156
Tertile 3	0.439 (0.18–1.06)	0.066	0.415 (0.17–1.02)	0.057	0.334 (0.12–0.93)	**0.037**

Model 1—crude; Model 2—adjusted for age and BMI; Model 3—additional adjustment for educational level, employment status, use of drugs, physical activity, family history of Alzheimer’s disease, genotype and depression.

**Table 5:** Association between antioxidant mineral intake and MoCA in a male population. Logistic regression analysis.

	Model 1		Model 2		Model 3	
	OR (95% CI)	*p*	OR (95% CI)	*p*	OR (95% CI)	*p*
**Iron**						
Tertile 1	1		1		1	
Tertile 2	0.851 (0.28–2.59)	0.777	0.966 (0.31–3.03)	0.952	1.075 (0.27–4.36)	0.919
Tertile 3	0.929 (0.30–2.86)	0.897	0.917 (0.29–2.89)	0.883	0.929 (0.22–3.90)	0.920
**Magnesium**						
Tertile 1	1		1		1	
Tertile 2	1.385 (0.45–4.25)	0.569	1.333 (0.41–4.30)	0.630	1.549 (0.38–6.21)	0.537
Tertile 3	0.923 (0.30–2.83)	0.889	0.982 (0.31–3.14)	0.975	1.189 (0.28–4.92)	0.811
**Copper**						
Tertile 1	1		1		1	
Tertile 2	0.413 (0.13–1.27)	0.124	0.338 (0.10–1.14)	0.080	0.261 (0.06–1.13)	0.070
Tertile 3	0.731 (0.23–2.33)	0.597	0.846 (0.25–2.84)	0.786	0.654 (0.14–2.91)	0.577
**Zinc**						
Tertile 1	1		1		1	
Tertile 2	0.609 (0.20–1.89)	0.391	0.593 (0.19–1.87)	0.373	0.351 (0.08–1.49)	0.157
Tertile 3	0.476 (0.15–1.50)	0.204	0.462 (0.14–1.50)	0.199	0.496 (0.13–1.93)	0.311
**Selenium**						
Tertile 1	1		1		1	
Tertile 2	1.385 (0.45–4.25)	0.569	1.634 (0.51–5.23)	0.409	1.195 (0.28–5.14)	0.811
Tertile 3	0.923 (0.30–2.83)	0.889	0.946 (0.30–2.98)	0.924	0.394 (0.09–1.68)	0.208
**Manganese**						
Tertile 1	1		1		1	
Tertile 2	0.617 (0.20–1.89)	0.397	0.656 (0.21–2.10)	0.478	0.448 (0.10–1.94)	0.283
Tertile 3	1.310 (0.42–4.11)	0.644	1.310 (0.40–4.33)	0.658	1.615 (0.35–7.29)	0.533

Model 1—crude; Model 2—adjusted for age and BMI; Model 3—additional adjustment for educational level, employment status, drugs, physical activity, family history of Alzheimer Disease, genotype and depression.

## Data Availability

The data presented in this study are available upon request from the corresponding author.
